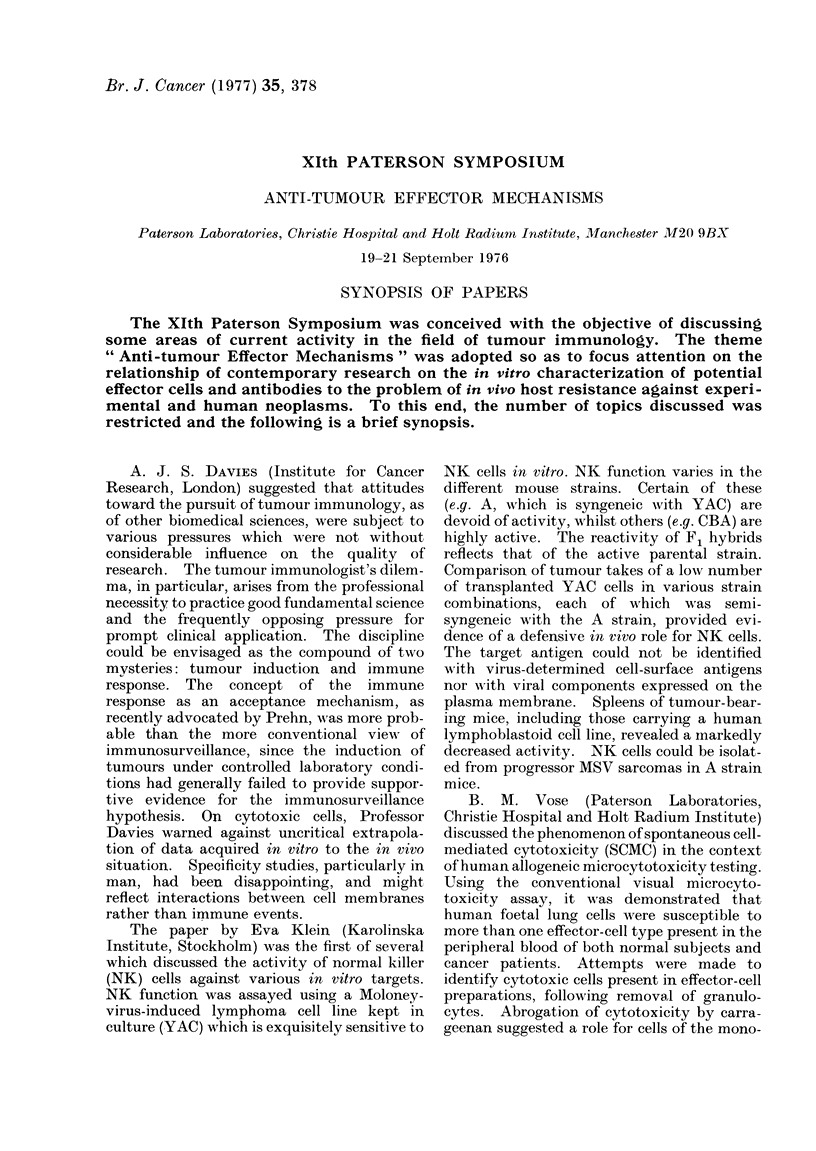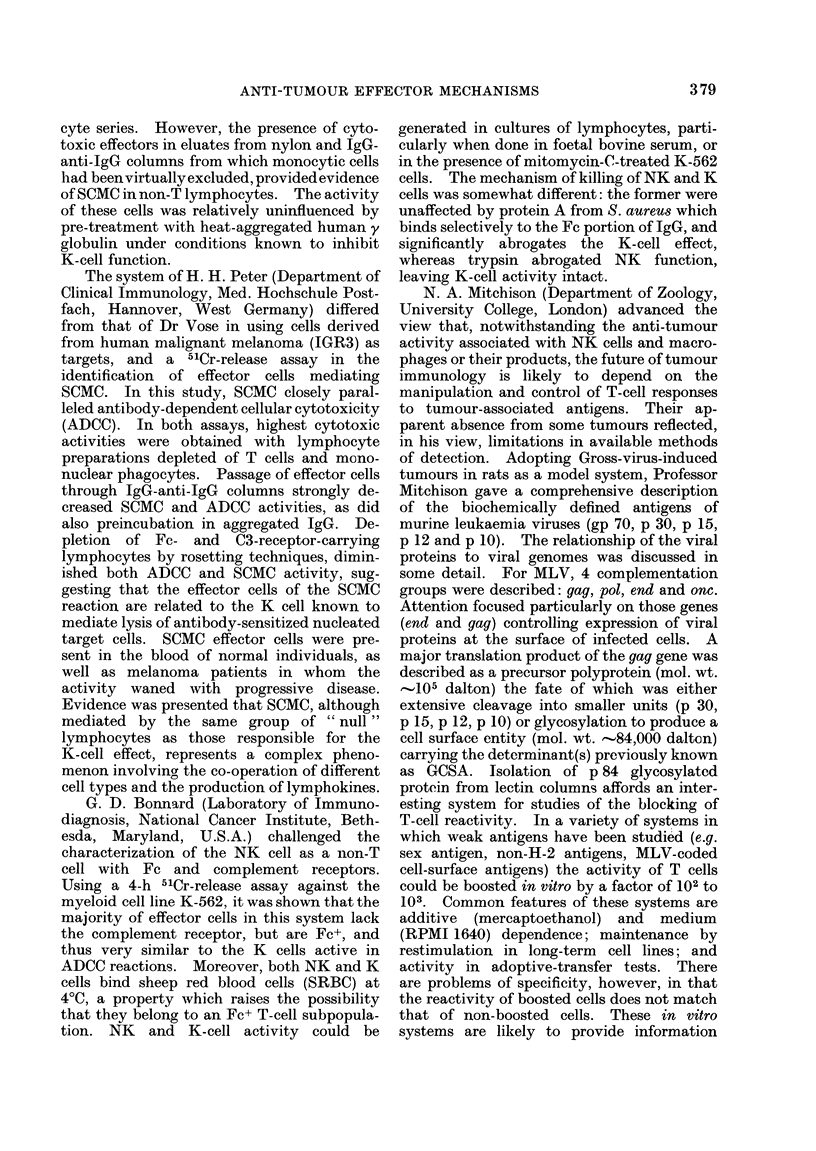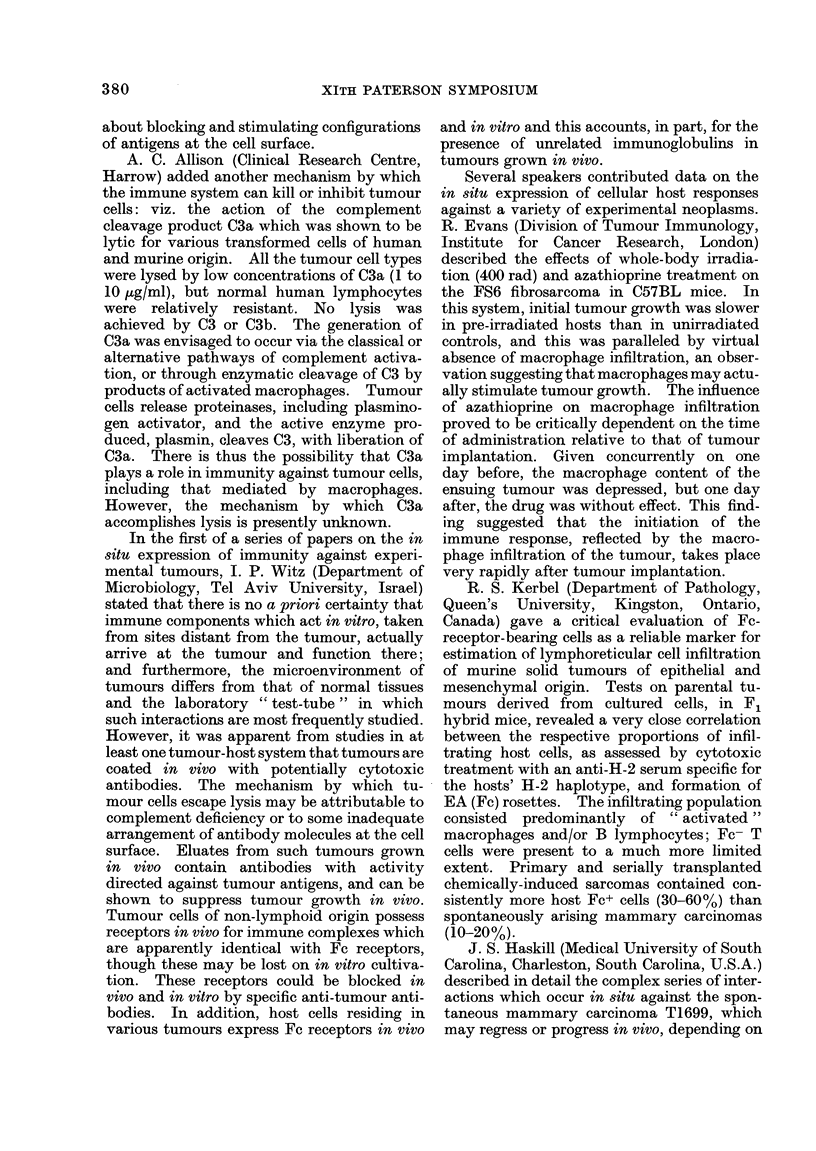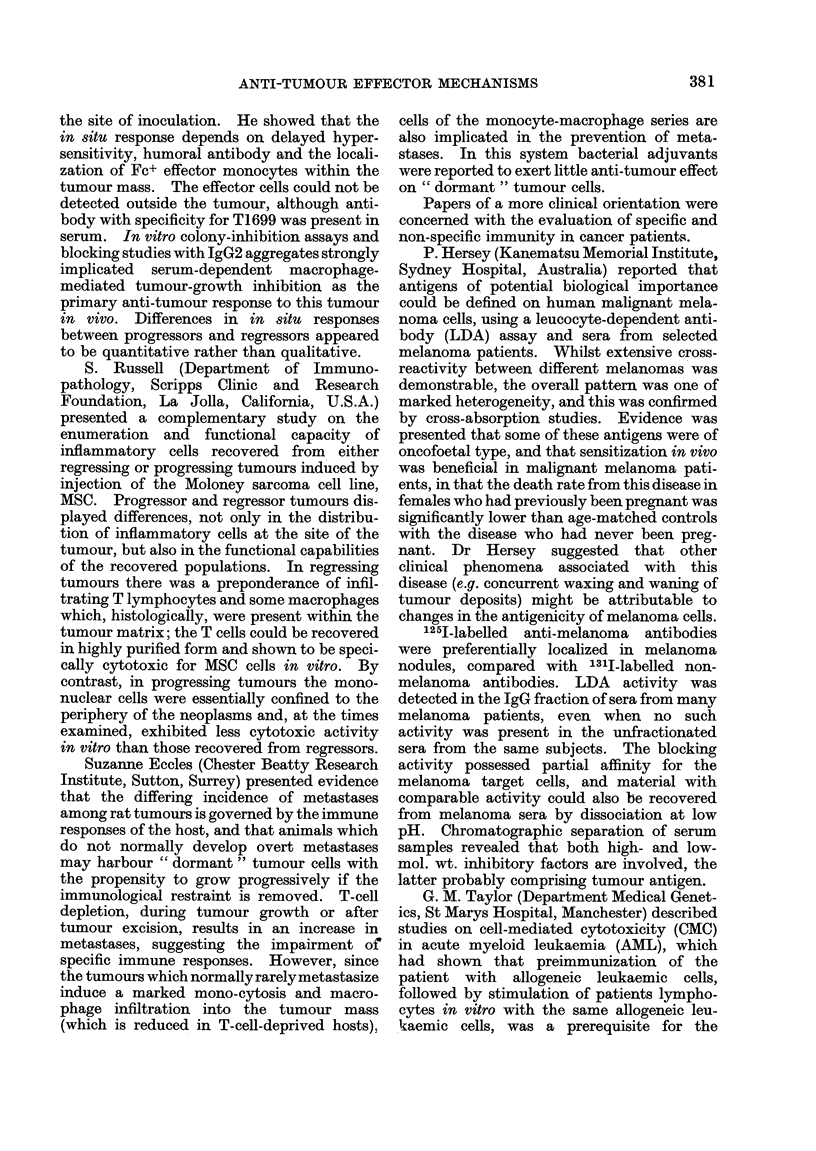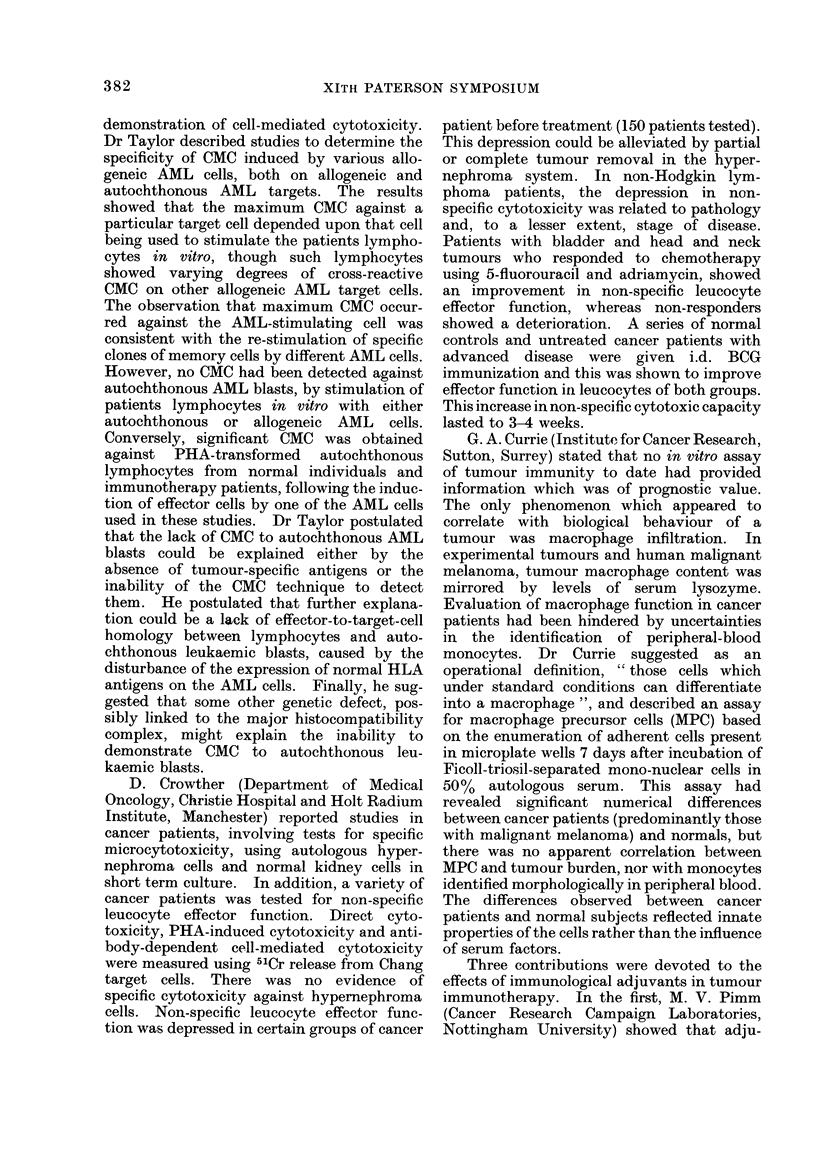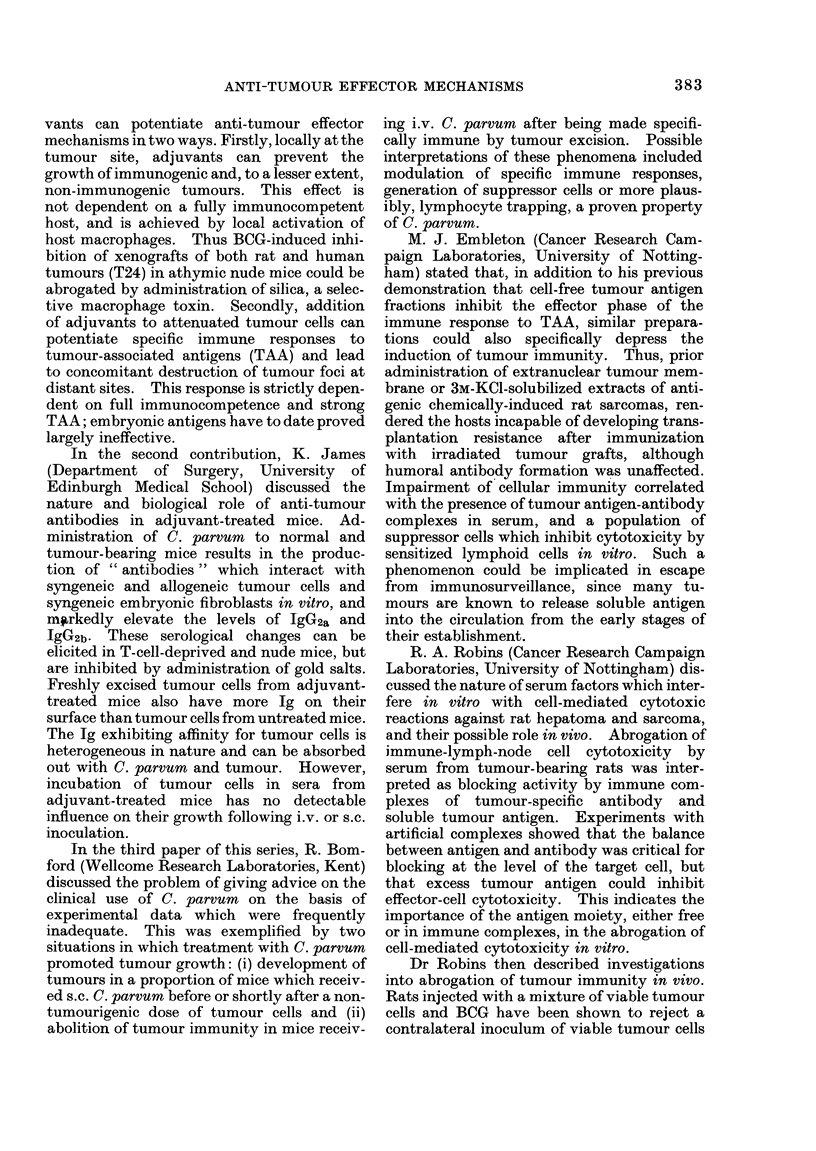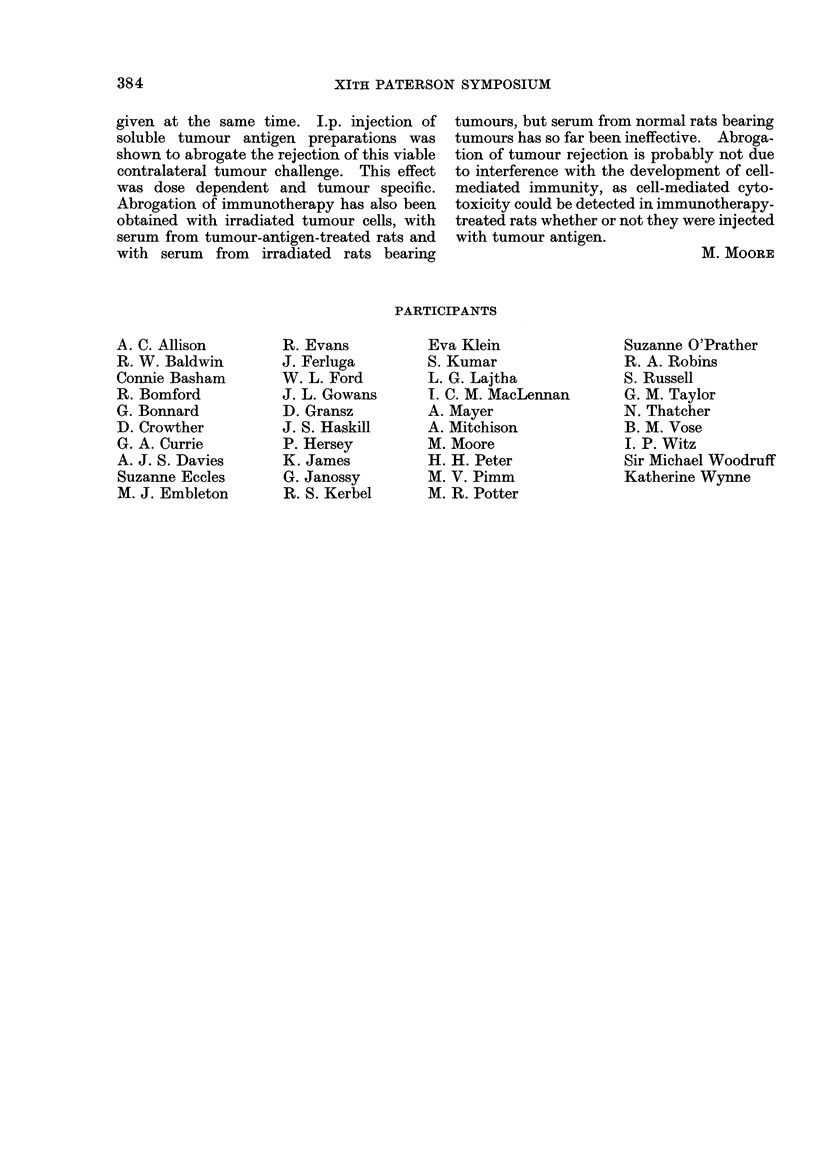# XIth Paterson Symposium

**Published:** 1977-03

**Authors:** M. Moore


*Br. J. Cancer* (1977) **35**, 378

### XIth PATERSON SYMPOSIUM

### ANTI-TUMOUR EFFECTOR MECHANISMS

*Paterson Laboratories, Christie Hospital and Holt Radium Institute, Manchester M20 9BX*

19–21 September 1976

### SYNOPSIS OF PAPERS

**The XIth Paterson Symposium was conceived with the objective of discussing some areas of current activity in the field of tumour immunology. The theme " Anti-tumour Effector Mechanisms " was adopted so as to focus attention on the relationship of contemporary research on the *in vitro* characterization of potential effector cells and antibodies to the problem of *in vivo* host resistance against experimental and human neoplasms. To this end, the number of topics discussed was restricted and the following is a brief synopsis.**

A. J. S. Davies (Institute for Cancer Research, London) suggested that attitudes toward the pursuit of tumour immunology, as of other biomedical sciences, were subject to various pressures which were not without considerable influence on the quality of research. The tumour immunologist's dilemma, in particular, arises from the professional necessity to practice good fundamental science and the frequently opposing pressure for prompt clinical application. The discipline could be envisaged as the compound of two mysteries: tumour induction and immune response. The concept of the immune response as an acceptance mechanism, as recently advocated by Prehn, was more probable than the more conventional view of immunosurveillance, since the induction of tumours under controlled laboratory conditions had generally failed to provide supportive evidence for the immunosurveillance hypothesis. On cytotoxic cells, Professor Davies warned against uncritical extrapolation of data acquired *in vitro* to the *in vivo* situation. Specificity studies, particularly in man, had been disappointing, and might reflect interactions between cell membranes rather than immune events.

The paper by Eva Klein (Karolinska Institute, Stockholm) was the first of several which discussed the activity of normal killer (NK) cells against various *in vitro* targets. NK function was assayed using a Moloney-virus-induced lymphoma cell line kept in culture (YAC) which is exquisitely sensitive to NK cells *in vitro*. NK function varies in the different mouse strains. Certain of these (*e.g.* A, which is syngeneic with YAC) are devoid of activity, whilst others (*e.g.* CBA) are highly active. The reactivity of $F_1$ hybrids reflects that of the active parental strain. Comparison of tumour takes of a low number of transplanted YAC cells in various strain combinations, each of which was semi-syngeneic with the A strain, provided evidence of a defensive *in vivo* role for NK cells. The target antigen could not be identified with virus-determined cell-surface antigens nor with viral components expressed on the plasma membrane. Spleens of tumour-bearing mice, including those carrying a human lymphoblastoid cell line, revealed a markedly decreased activity. NK cells could be isolated from progressor MSV sarcomas in A strain mice.

B. M. Vose (Paterson Laboratories, Christie Hospital and Holt Radium Institute) discussed the phenomenon of spontaneous cell-mediated cytotoxicity (SCMC) in the context of human allogeneic microcytotoxicity testing. Using the conventional visual microcytotoxicity assay, it was demonstrated that human foetal lung cells were susceptible to more than one effector-cell type present in the peripheral blood of both normal subjects and cancer patients. Attempts were made to identify cytotoxic cells present in effector-cell preparations, following removal of granulocytes. Abrogation of cytotoxicity by carrageenan suggested a role for cells of the monocyte series. However, the presence of cytotoxic effectors in eluates from nylon and IgG-anti-IgG columns from which monocytic cells had been virtually excluded, provided evidence of SCMC in non-T lymphocytes. The activity of these cells was relatively uninfluenced by pre-treatment with heat-aggregated human $\gamma$ globulin under conditions known to inhibit K-cell function.

The system of H. H. Peter (Department of Clinical Immunology, Med. Hochschule Postfach, Hannover, West Germany) differed from that of Dr Vose in using cells derived from human malignant melanoma (IGR3) as targets, and a $^{51}$Cr-release assay in the identification of effector cells mediating SCMC. In this study, SCMC closely paralleled antibody-dependent cellular cytotoxicity (ADCC). In both assays, highest cytotoxic activities were obtained with lymphocyte preparations depleted of T cells and mononuclear phagocytes. Passage of effector cells through IgG-anti-IgG columns strongly decreased SCMC and ADCC activities, as did also preincubation in aggregated IgG. Depletion of Fc- and C3-receptor-carrying lymphocytes by rosetting techniques, diminished both ADCC and SCMC activity, suggesting that the effector cells of the SCMC reaction are related to the K cell known to mediate lysis of antibody-sensitized nucleated target cells. SCMC effector cells were present in the blood of normal individuals, as well as melanoma patients in whom the activity waned with progressive disease. Evidence was presented that SCMC, although mediated by the same group of "null" lymphocytes as those responsible for the K-cell effect, represents a complex phenomenon involving the co-operation of different cell types and the production of lymphokines.

G. D. Bonnard (Laboratory of Immunodiagnosis, National Cancer Institute, Bethesda, Maryland, U.S.A.) challenged the characterization of the NK cell as a non-T cell with Fc and complement receptors. Using a 4-h $^{51}$Cr-release assay against the myeloid cell line K-562, it was shown that the majority of effector cells in this system lack the complement receptor, but are Fc$^{+}$, and thus very similar to the K cells active in ADCC reactions. Moreover, both NK and K cells bind sheep red blood cells (SRBC) at 4°C, a property which raises the possibility that they belong to an Fc$^{+}$ T-cell subpopulation. NK and K-cell activity could be generated in cultures of lymphocytes, particularly when done in foetal bovine serum, or in the presence of mitomycin-C-treated K-562 cells. The mechanism of killing of NK and K cells was somewhat different: the former were unaffected by protein A from *S. aureus* which binds selectively to the Fc portion of IgG, and significantly abrogates the K-cell effect, whereas trypsin abrogated NK function, leaving K-cell activity intact.

N. A. Mitchison (Department of Zoology, University College, London) advanced the view that, notwithstanding the anti-tumour activity associated with NK cells and macrophages or their products, the future of tumour immunology is likely to depend on the manipulation and control of T-cell responses to tumour-associated antigens. Their apparent absence from some tumours reflected, in his view, limitations in available methods of detection. Adopting Gross-virus-induced tumours in rats as a model system, Professor Mitchison gave a comprehensive description of the biochemically defined antigens of murine leukaemia viruses (gp 70, p 30, p 15, p 12 and p 10). The relationship of the viral proteins to viral genomes was discussed in some detail. For MLV, 4 complementation groups were described: *gag*, *pol*, *end* and *onc*. Attention focused particularly on those genes (*end* and *gag*) controlling expression of viral proteins at the surface of infected cells. A major translation product of the *gag* gene was described as a precursor polyprotein (mol. wt. $\sim 10^5$ dalton) the fate of which was either extensive cleavage into smaller units (p 30, p 15, p 12, p 10) or glycosylation to produce a cell surface entity (mol. wt. $\sim$84,000 dalton) carrying the determinant(s) previously known as GCSA. Isolation of p 84 glycosylated protein from lectin columns affords an interesting system for studies of the blocking of T-cell reactivity. In a variety of systems in which weak antigens have been studied (*e.g.* sex antigen, non-H-2 antigens, MLV-coded cell-surface antigens) the activity of T cells could be boosted *in vitro* by a factor of $10^2$ to $10^3$. Common features of these systems are additive (mercaptoethanol) and medium (RPMI 1640) dependence; maintenance by restimulation in long-term cell lines; and activity in adoptive-transfer tests. There are problems of specificity, however, in that the reactivity of boosted cells does not match that of non-boosted cells. These *in vitro* systems are likely to provide information about blocking and stimulating configurations of antigens at the cell surface.

A. C. Allison (Clinical Research Centre, Harrow) added another mechanism by which the immune system can kill or inhibit tumour cells: viz. the action of the complement cleavage product C3a which was shown to be lytic for various transformed cells of human and murine origin. All the tumour cell types were lysed by low concentrations of C3a (1 to 10 $\mu$g/ml), but normal human lymphocytes were relatively resistant. No lysis was achieved by C3 or C3b. The generation of C3a was envisaged to occur via the classical or alternative pathways of complement activation, or through enzymatic cleavage of C3 by products of activated macrophages. Tumour cells release proteinases, including plasminogen activator, and the active enzyme produced, plasmin, cleaves C3, with liberation of C3a. There is thus the possibility that C3a plays a role in immunity against tumour cells, including that mediated by macrophages. However, the mechanism by which C3a accomplishes lysis is presently unknown.

In the first of a series of papers on the *in situ* expression of immunity against experimental tumours, I. P. Witz (Department of Microbiology, Tel Aviv University, Israel) stated that there is no *a priori* certainty that immune components which act *in vitro*, taken from sites distant from the tumour, actually arrive at the tumour and function there; and furthermore, the microenvironment of tumours differs from that of normal tissues and the laboratory "test-tube" in which such interactions are most frequently studied. However, it was apparent from studies in at least one tumour-host system that tumours are coated *in vivo* with potentially cytotoxic antibodies. The mechanism by which tumour cells escape lysis may be attributable to complement deficiency or to some inadequate arrangement of antibody molecules at the cell surface. Eluates from such tumours grown *in vivo* contain antibodies with activity directed against tumour antigens, and can be shown to suppress tumour growth *in vivo*. Tumour cells of non-lymphoid origin possess receptors *in vivo* for immune complexes which are apparently identical with Fc receptors, though these may be lost on *in vitro* cultivation. These receptors could be blocked *in vivo* and *in vitro* by specific anti-tumour antibodies. In addition, host cells residing in various tumours express Fc receptors *in vivo* and *in vitro* and this accounts, in part, for the presence of unrelated immunoglobulins in tumours grown *in vivo*.

Several speakers contributed data on the *in situ* expression of cellular host responses against a variety of experimental neoplasms. R. Evans (Division of Tumour Immunology, Institute for Cancer Research, London) described the effects of whole-body irradiation (400 rad) and azathioprine treatment on the FS6 fibrosarcoma in C57BL mice. In this system, initial tumour growth was slower in pre-irradiated hosts than in unirradiated controls, and this was paralleled by virtual absence of macrophage infiltration, an observation suggesting that macrophages may actually stimulate tumour growth. The influence of azathioprine on macrophage infiltration proved to be critically dependent on the time of administration relative to that of tumour implantation. Given concurrently on one day before, the macrophage content of the ensuing tumour was depressed, but one day after, the drug was without effect. This finding suggested that the initiation of the immune response, reflected by the macrophage infiltration of the tumour, takes place very rapidly after tumour implantation.

R. S. Kerbel (Department of Pathology, Queen's University, Kingston, Ontario, Canada) gave a critical evaluation of Fc-receptor-bearing cells as a reliable marker for estimation of lymphoreticular cell infiltration of murine solid tumours of epithelial and mesenchymal origin. Tests on parental tumours derived from cultured cells, in $F_1$ hybrid mice, revealed a very close correlation between the respective proportions of infiltrating host cells, as assessed by cytotoxic treatment with an anti-H-2 serum specific for the hosts' H-2 haplotype, and formation of EA (Fc) rosettes. The infiltrating population consisted predominantly of "activated" macrophages and/or B lymphocytes; $Fc^-$ T cells were present to a much more limited extent. Primary and serially transplanted chemically-induced sarcomas contained consistently more host $Fc^+$ cells (30–60%) than spontaneously arising mammary carcinomas (10–20%).

J. S. Haskill (Medical University of South Carolina, Charleston, South Carolina, U.S.A.) described in detail the complex series of interactions which occur *in situ* against the spontaneous mammary carcinoma T1699, which may regress or progress *in vivo*, depending on the site of inoculation. He showed that the *in situ* response depends on delayed hypersensitivity, humoral antibody and the localization of $Fc^+$ effector monocytes within the tumour mass. The effector cells could not be detected outside the tumour, although antibody with specificity for T1699 was present in serum. *In vitro* colony-inhibition assays and blocking studies with IgG2 aggregates strongly implicated serum-dependent macrophage-mediated tumour-growth inhibition as the primary anti-tumour response to this tumour *in vivo*. Differences in *in situ* responses between progressors and regressors appeared to be quantitative rather than qualitative.

S. Russell (Department of Immunopathology, Scripps Clinic and Research Foundation, La Jolla, California, U.S.A.) presented a complementary study on the enumeration and functional capacity of inflammatory cells recovered from either regressing or progressing tumours induced by injection of the Moloney sarcoma cell line, MSC. Progressor and regressor tumours displayed differences, not only in the distribution of inflammatory cells at the site of the tumour, but also in the functional capabilities of the recovered populations. In regressing tumours there was a preponderance of infiltrating T lymphocytes and some macrophages which, histologically, were present within the tumour matrix; the T cells could be recovered in highly purified form and shown to be specically cytotoxic for MSC cells *in vitro*. By contrast, in progressing tumours the mononuclear cells were essentially confined to the periphery of the neoplasms and, at the times examined, exhibited less cytotoxic activity *in vitro* than those recovered from regressors.

Suzanne Eccles (Chester Beatty Research Institute, Sutton, Surrey) presented evidence that the differing incidence of metastases among rat tumours is governed by the immune responses of the host, and that animals which do not normally develop overt metastases may harbour " dormant " tumour cells with the propensity to grow progressively if the immunological restraint is removed. T-cell depletion, during tumour growth or after tumour excision, results in an increase in metastases, suggesting the impairment of specific immune responses. However, since the tumours which normally rarely metastasize induce a marked mono-cytosis and macrophage infiltration into the tumour mass (which is reduced in T-cell-deprived hosts), cells of the monocyte-macrophage series are also implicated in the prevention of metastases. In this system bacterial adjuvants were reported to exert little anti-tumour effect on " dormant " tumour cells.

Papers of a more clinical orientation were concerned with the evaluation of specific and non-specific immunity in cancer patients.

P. Hersey (Kanematsu Memorial Institute, Sydney Hospital, Australia) reported that antigens of potential biological importance could be defined on human malignant melanoma cells, using a leucocyte-dependent antibody (LDA) assay and sera from selected melanoma patients. Whilst extensive cross-reactivity between different melanomas was demonstrable, the overall pattern was one of marked heterogeneity, and this was confirmed by cross-absorption studies. Evidence was presented that some of these antigens were of oncofoetal type, and that sensitization *in vivo* was beneficial in malignant melanoma patients, in that the death rate from this disease in females who had previously been pregnant was significantly lower than age-matched controls with the disease who had never been pregnant. Dr Hersey suggested that other clinical phenomena associated with this disease (*e.g.* concurrent waxing and waning of tumour deposits) might be attributable to changes in the antigenicity of melanoma cells.

$^{125}$I-labelled anti-melanoma antibodies were preferentially localized in melanoma nodules, compared with $^{131}$I-labelled non-melanoma antibodies. LDA activity was detected in the IgG fraction of sera from many melanoma patients, even when no such activity was present in the unfractionated sera from the same subjects. The blocking activity possessed partial affinity for the melanoma target cells, and material with comparable activity could also be recovered from melanoma sera by dissociation at low pH. Chromatographic separation of serum samples revealed that both high- and low-mol. wt. inhibitory factors are involved, the latter probably comprising tumour antigen.

G. M. Taylor (Department Medical Genetics, St Marys Hospital, Manchester) described studies on cell-mediated cytotoxicity (CMC) in acute myeloid leukaemia (AML), which had shown that preimmunization of the patient with allogeneic leukaemic cells, followed by stimulation of patients lymphocytes *in vitro* with the same allogeneic leukaemic cells, was a prerequisite for the demonstration of cell-mediated cytotoxicity. Dr Taylor described studies to determine the specificity of CMC induced by various allogeneic AML cells, both on allogeneic and autochthonous AML targets. The results showed that the maximum CMC against a particular target cell depended upon that cell being used to stimulate the patients lymphocytes *in vitro*, though such lymphocytes showed varying degrees of cross-reactive CMC on other allogeneic AML target cells. The observation that maximum CMC occurred against the AML-stimulating cell was consistent with the re-stimulation of specific clones of memory cells by different AML cells. However, no CMC had been detected against autochthonous AML blasts, by stimulation of patients lymphocytes *in vitro* with either autochthonous or allogeneic AML cells. Conversely, significant CMC was obtained against PHA-transformed autochthonous lymphocytes from normal individuals and immunotherapy patients, following the induction of effector cells by one of the AML cells used in these studies. Dr Taylor postulated that the lack of CMC to autochthonous AML blasts could be explained either by the absence of tumour-specific antigens or the inability of the CMC technique to detect them. He postulated that further explanation could be a lack of effector-to-target-cell homology between lymphocytes and autochthonous leukaemic blasts, caused by the disturbance of the expression of normal HLA antigens on the AML cells. Finally, he suggested that some other genetic defect, possibly linked to the major histocompatibility complex, might explain the inability to demonstrate CMC to autochthonous leukaemic blasts.

D. Crowther (Department of Medical Oncology, Christie Hospital and Holt Radium Institute, Manchester) reported studies in cancer patients, involving tests for specific microcytotoxicity, using autologous hypernephroma cells and normal kidney cells in short term culture. In addition, a variety of cancer patients was tested for non-specific leucocyte effector function. Direct cytotoxicity, PHA-induced cytotoxicity and antibody-dependent cell-mediated cytotoxicity were measured using $^{51}Cr$ release from Chang target cells. There was no evidence of specific cytotoxicity against hypernephroma cells. Non-specific leucocyte effector function was depressed in certain groups of cancer patient before treatment (150 patients tested). This depression could be alleviated by partial or complete tumour removal in the hypernephroma system. In non-Hodgkin lymphoma patients, the depression in non-specific cytotoxicity was related to pathology and, to a lesser extent, stage of disease. Patients with bladder and head and neck tumours who responded to chemotherapy using 5-fluorouracil and adriamycin, showed an improvement in non-specific leucocyte effector function, whereas non-responders showed a deterioration. A series of normal controls and untreated cancer patients with advanced disease were given i.d. BCG immunization and this was shown to improve effector function in leucocytes of both groups. This increase in non-specific cytotoxic capacity lasted to 3–4 weeks.

G. A. Currie (Institute for Cancer Research, Sutton, Surrey) stated that no *in vitro* assay of tumour immunity to date had provided information which was of prognostic value. The only phenomenon which appeared to correlate with biological behaviour of a tumour was macrophage infiltration. In experimental tumours and human malignant melanoma, tumour macrophage content was mirrored by levels of serum lysozyme. Evaluation of macrophage function in cancer patients had been hindered by uncertainties in the identification of peripheral-blood monocytes. Dr Currie suggested as an operational definition, " those cells which under standard conditions can differentiate into a macrophage ", and described an assay for macrophage precursor cells (MPC) based on the enumeration of adherent cells present in microplate wells 7 days after incubation of Ficoll-triosil-separated mono-nuclear cells in 50% autologous serum. This assay had revealed significant numerical differences between cancer patients (predominantly those with malignant melanoma) and normals, but there was no apparent correlation between MPC and tumour burden, nor with monocytes identified morphologically in peripheral blood. The differences observed between cancer patients and normal subjects reflected innate properties of the cells rather than the influence of serum factors.

Three contributions were devoted to the effects of immunological adjuvants in tumour immunotherapy. In the first, M. V. Pimm (Cancer Research Campaign Laboratories, Nottingham University) showed that adjuvants can potentiate anti-tumour effector mechanisms in two ways. Firstly, locally at the tumour site, adjuvants can prevent the growth of immunogenic and, to a lesser extent, non-immunogenic tumours. This effect is not dependent on a fully immunocompetent host, and is achieved by local activation of host macrophages. Thus BCG-induced inhibition of xenografts of both rat and human tumours (T24) in athymic nude mice could be abrogated by administration of silica, a selective macrophage toxin. Secondly, addition of adjuvants to attenuated tumour cells can potentiate specific immune responses to tumour-associated antigens (TAA) and lead to concomitant destruction of tumour foci at distant sites. This response is strictly dependent on full immunocompetence and strong TAA; embryonic antigens have to date proved largely ineffective.

In the second contribution, K. James (Department of Surgery, University of Edinburgh Medical School) discussed the nature and biological role of anti-tumour antibodies in adjuvant-treated mice. Administration of *C. parvum* to normal and tumour-bearing mice results in the production of "antibodies" which interact with syngeneic and allogeneic tumour cells and syngeneic embryonic fibroblasts *in vitro*, and markedly elevate the levels of $IgG_{2a}$ and $IgG_{2b}$. These serological changes can be elicited in T-cell-deprived and nude mice, but are inhibited by administration of gold salts. Freshly excised tumour cells from adjuvant-treated mice also have more Ig on their surface than tumour cells from untreated mice. The Ig exhibiting affinity for tumour cells is heterogeneous in nature and can be absorbed out with *C. parvum* and tumour. However, incubation of tumour cells in sera from adjuvant-treated mice has no detectable influence on their growth following i.v. or s.c. inoculation.

In the third paper of this series, R. Bomford (Wellcome Research Laboratories, Kent) discussed the problem of giving advice on the clinical use of *C. parvum* on the basis of experimental data which were frequently inadequate. This was exemplified by two situations in which treatment with *C. parvum* promoted tumour growth: (i) development of tumours in a proportion of mice which received s.c. *C. parvum* before or shortly after a non-tumourigenic dose of tumour cells and (ii) abolition of tumour immunity in mice receiving i.v. *C. parvum* after being made specifically immune by tumour excision. Possible interpretations of these phenomena included modulation of specific immune responses, generation of suppressor cells or more plausibly, lymphocyte trapping, a proven property of *C. parvum*.

M. J. Embleton (Cancer Research Campaign Laboratories, University of Nottingham) stated that, in addition to his previous demonstration that cell-free tumour antigen fractions inhibit the effector phase of the immune response to TAA, similar preparations could also specifically depress the induction of tumour immunity. Thus, prior administration of extranuclear tumour membrane or 3M-KCl-solubilized extracts of antigenic chemically-induced rat sarcomas, rendered the hosts incapable of developing transplantation resistance after immunization with irradiated tumour grafts, although humoral antibody formation was unaffected. Impairment of cellular immunity correlated with the presence of tumour antigen-antibody complexes in serum, and a population of suppressor cells which inhibit cytotoxicity by sensitized lymphoid cells *in vitro*. Such a phenomenon could be implicated in escape from immunosurveillance, since many tumours are known to release soluble antigen into the circulation from the early stages of their establishment.

R. A. Robins (Cancer Research Campaign Laboratories, University of Nottingham) discussed the nature of serum factors which interfere *in vitro* with cell-mediated cytotoxic reactions against rat hepatoma and sarcoma, and their possible role *in vivo*. Abrogation of immune-lymph-node cell cytotoxicity by serum from tumour-bearing rats was interpreted as blocking activity by immune complexes of tumour-specific antibody and soluble tumour antigen. Experiments with artificial complexes showed that the balance between antigen and antibody was critical for blocking at the level of the target cell, but that excess tumour antigen could inhibit effector-cell cytotoxicity. This indicates the importance of the antigen moiety, either free or in immune complexes, in the abrogation of cell-mediated cytotoxicity *in vitro*.

Dr Robins then described investigations into abrogation of tumour immunity *in vivo*. Rats injected with a mixture of viable tumour cells and BCG have been shown to reject a contralateral inoculum of viable tumour cells given at the same time. I.p. injection of soluble tumour antigen preparations was shown to abrogate the rejection of this viable contralateral tumour challenge. This effect was dose dependent and tumour specific. Abrogation of immunotherapy has also been obtained with irradiated tumour cells, with serum from tumour-antigen-treated rats and with serum from irradiated rats bearing tumours, but serum from normal rats bearing tumours has so far been ineffective. Abrogation of tumour rejection is probably not due to interference with the development of cell-mediated immunity, as cell-mediated cytotoxicity could be detected in immunotherapy-treated rats whether or not they were injected with tumour antigen.

M. Moore

PARTICIPANTS

A. C. Allison
R. W. Baldwin
Connie Basham
R. Bomford
G. Bonnard
D. Crowther
G. A. Currie
A. J. S. Davies
Suzanne Eccles
M. J. Embleton
R. Evans
J. Ferluga
W. L. Ford
J. L. Gowans
D. Gransz
J. S. Haskill
P. Hersey
K. James
G. Janossy
R. S. Kerbel
Eva Klein
S. Kumar
L. G. Lajtha
I. C. M. MacLennan
A. Mayer
A. Mitchison
M. Moore
H. H. Peter
M. V. Pimm
M. R. Potter
Suzanne O'Prather
R. A. Robins
S. Russell
G. M. Taylor
N. Thatcher
B. M. Vose
I. P. Witz
Sir Michael Woodruff
Katherine Wynne